# Coronary cytoskeletal modulation of coronary blood flow in the presence and absence of type 2 diabetes: the role of cofilin

**DOI:** 10.3389/fphys.2025.1561867

**Published:** 2025-03-18

**Authors:** Patricia E. McCallinhart, Kathlyene R. Stone, Pamela A. Lucchesi, Aaron J. Trask

**Affiliations:** ^1^ Center for Cardiovascular Research, The Heart Center, The Abigail Wexner Research Institute at Nationwide Children’s Hospital, Columbus, OH, United States; ^2^ Department of Undergraduate Medical Education, University of Texas Tyler School of Medicine, Tyler, TX, United States; ^3^ Department of Pediatrics, The Ohio State University College of Medicine, Columbus, OH, United States

**Keywords:** coronary microcirculation, vascular smooth muscle cells, diabetes, cofilin, actin remodeling, coronary blood flow

## Abstract

**Background:**

Coronary resistance microvessels (CRMs) from type 2 diabetic (T2DM) mice and pigs are less stiff compared to normal, a finding that is dictated by less stiff coronary vascular smooth muscle cells (VSMCs). Cofilin is an endogenous actin regulatory protein that depolymerizes filamentous (F)-actin, and portions of F-actin bound to cofilin are less stiff compared to their unbound F-actin counterparts. In this study, we hypothesized that altering the actin cytoskeleton modifies VSMC stiffness, which contributes to changes in coronary blood flow in normal and T2DM conditions.

**Methods and results:**

Utilizing phalloidin staining, we found that F-actin was significantly reduced in T2DM CRM VSMCs, and we showed cofilin expression was increased in T2DM by proteomics and Western blot analysis. Cofilin knockdown in both human and mouse coronary VSMCs using siRNA significantly increased F/G actin ratio. Cofilin knockdown also caused a significant increase in elastic modulus by atomic force microscopy of coronary VSMCs. Treatment with Latrunculin B, an actin disruptor, significantly decreased VSMC elastic modulus. Acute Latrunculin B infusion into the coronary circulation of *ex vivo* isolated Langendorff mouse hearts increased peak coronary blood flow.

**Conclusion:**

Together, we demonstrated that the CRM VSMC actin cytoskeleton is altered in T2DM to favor less stiff cells, and pharmacological manipulation of the actin cytoskeleton alters VSMC biomechanics. This study is also the first to demonstrate that coronary cellular modulation of mechanics can acutely modulate coronary blood flow.

## 1 Introduction

Type 2 diabetes mellitus (T2DM) is a metabolic disorder characterized by hyperglycemia and insulin resistance and is recognized as a cardiovascular disease due to the high associated prevalence of myocardial infarction (MI), atherosclerosis, and coronary artery disease (CAD) in T2DM patients (Gil-Ortega and Carlos Kaski, 2006; Mozaffarian et al., 2015). Over 500 million adults between the ages 20–79 are living with diabetes and it is projected to reach 643 million by 2030 and 783 million by 2045 (IDF Diabetes Atlas, 2023). Despite several decades of research and therapy targeting these conditions, cardiovascular complications, including MI, remain the number one cause of death in T2DM patients (Tsao et al., 2023; Grundy et al., 1999). Coronary blood flow (CBF) is crucial for supplying the heart cells with the nutrients they need to meet their metabolic demands, and any reduction in CBF can lead to severe conditions like heart failure. Coronary microvascular disease (CMD) is increasingly recognized as an early subclinical contributor to perturbations in CBF and ultimately, heart disease (McCallinhart et al., 2024). Previous data from our laboratory demonstrated that adverse structural remodeling of coronary resistance microvessels (CRMs) and impaired CBF occur in T2DM (Katz et al., 2011; Trask et al., 2012a; Trask et al., 2012b; Husarek et al., 2016a; Trask et al., 2012a). We previously reported that the process of T2DM CRM remodeling occurs early, equivalent to premature aging (McCallinhart et al., 2018). Diabetic macrovessels such as the aorta and carotid arteries increase in stiffness (Reddy, 2004). However, previous anti-dogmatic data from our laboratory revealed that CRMs are less stiff in both T2DM and metabolic syndrome (MetS) (Katz et al., 2011; Trask et al., 2012b) – a finding that does not appear to be due to the extracellular matrix (ECM) (Anghelescu et al., 2015; Katz et al., 2011; Trask et al., 2012a). Rather, utilizing atomic force microscopy (AFM), we showed that primary CRM vascular smooth muscle cells (VSMCs) isolated from db/db mice and T2DM humans were less stiff relative to normal, indicating that overall CRM tissue stiffness is driven by coronary VSMCs (McCallinhart et al., 2020). This observation raised the intriguing possibility that the molecular mechanisms that regulate cellular stiffness may play a central role in the pathophysiology of CMD.

The VSMC actin cytoskeleton is an integral component that not only contributes to the structural integrity of the cell, but also allows for cell movement and contraction. The connection of cytoskeletal components to the basement membrane and the surrounding ECM is dictated by focal adhesion (FA) complexes and together they contribute to cellular mechanics (Lacolley et al., 2017). Actin’s structural and biomechanical roles also arise from the ability to dynamically polymerize and depolymerize, forming polymers and filaments of varying lengths (Serebryannyy et al., 2016). Filamentous actin (F-actin) is a thin flexible fiber-type microfilament made up of monomeric globular actin (G-actin). It is widely accepted that actin filaments provide mechanical stability by regulating cell elastic modulus (Fels et al., 2014; Grimm et al., 2014; Sehgel et al., 2013) and that disruption of the cytoskeleton via pharmacological agents causes a striking reduction in cell stiffness (Petersen et al., 1982; Wakatsuki et al., 2001). Conversely, actin stabilizers/polymerizers, including jasplakinolide, enhance myogenic tone via smooth muscle contraction and increased F-actin to G actin-ratio (Cipolla et al., 2002).

Cofilin is an endogenous actin regulatory protein that depolymerizes F-actin and severs the actin filaments. Interestingly, portions of F-actin bound to cofilin have decreased stiffness compared to their unbound F-actin counterparts (McCullough et al., 2008; Schramm et al., 2017). Interestingly, there are reports that arterial stiffening adversely affects coronary flow and flow reserve, but those data are taken from large conduit arteries outside of the coronary circulation (Fukuda et al., 2006; Leung et al., 2006; Tritakis et al., 2016). However, little is known about the role of altering the actin cytoskeleton in coronary VSMCs and how that contributes to CBF. The goal of this present study was to test the hypothesis that modulating coronary cellular stiffness inversely affects coronary blood flow in normal and T2DM mice and that cofilin plays a role. To test this hypothesis, we used a combination of siRNA knockdown experiments, AFM, pharmacological modulation of the actin cytoskeleton, and Langendorff isolated hearts in mice.

## 2 Methods and materials

### 2.1 Materials

Antibodies: Total cofilin (D3F9) #5175 (concentration 1:1000) was acquired from Cell Signaling Technology for Western blot analysis. Beta actin (8226) (concentration 1:1000) was obtained from Abcam for Western blot analysis. Cofilin (PA5-27627) for staining (concentration 1:200), deoxyribonuclease 1 Alexa Fluor 488 for G-actin staining (concentration 1:200), and phalloidin Alexa Fluro 568 for F-actin staining (concentration 1:400), were all purchased from Invitrogen (Waltham, MA). 4′,6-Diamidino-2-phenylindole dihydrochloride (DAPI) from MP Biomedicals (Solon, OH) was used for nuclear staining (1:500).

Mouse cofilin siRNA and scramble siRNA were purchased from Invitrogen (Waltham, MA). Human cofilin siRNA and scramble were purchased from Dharmacon (Lafayette, CO). Deidentified normal and T2DM human primary coronary VSMCs were obtained from both Lonza (Morristown, NJ) and ATCC (Manassas, VA). All other reagents were purchase from Fisher Scientific (Pittsburgh, PA) unless otherwise noted.

### 2.2 Animals

Male T2DM homozygous *db*/*db* and control nondiabetic heterozygous *Db*/*db* mice were acquired from The Jackson Laboratories. By 4 weeks of age, *db*/*db* mice are leptin receptor deficient and develop hyperglycemia, obesity, insulin resistance, and dyslipidemia. They were housed under a 12 h light,12 h dark cycle at 22°C and 60% humidity and were allowed *ad libitum* access to standard low-fat laboratory chow and water. All experiments were conducted at 16–17 weeks of age. This study was conducted in accordance with National Institutes of Health guidelines, and it was approved by the Institutional Animal Care and Use Committee at Nationwide Children’s Hospital.

### 2.3 Blood glucose measurements

Blood was drawn from the mouse tail vein and glucose levels were determined with the AlphaTrak glucometer (Abbott Laboratories, Alameda, CA).

### 2.4 Proteomics

Septal CRMs were isolated from both normal heterozygous Db/db and diabetic homozygous db/db mice and were pooled (n = 4-5 pools of 4 CRMs per biological replicate) for proteomic data collection/analysis. CRMs were lysed by repeated sonication in 110 µL tissue lysis buffer + protease and phosphatase inhibitors + antifoaming agent. Lysates were kept cold and centrifuged briefly at 4°C between sonications to bring unlysed material to the bottom of the tube. All lysates were centrifuged 10 min, 4°C, 14,000 RPM and supernatants were removed to fresh tubes. 16 μg protein of each sample were methanol/chloroform precipitated. Dried samples were resuspended in SDS loading buffer, heated to 70°C for 5 min, and then separated on a 10% SDS-PAGE gel. The Sypro-stained gel was analyzed by LC-MS/MS (LTQ mass spec followed by 2D LC chromatography–MudPIT; Mascot sequence identification) at the Ohio State University Mass Spec & Proteomics Facility.

### 2.5 Mouse coronary VSMC isolation

Normal Db/db and T2DM db/db mouse CRM VSMCs were isolated as previously described by our laboratory (Husarek et al., 2016b; McCallinhart et al., 2020). In short, hearts were isolated from anesthetized mice (3% isoflurane until lack of toe pinch response) and gently perfused via retrograde Langendorff with digestion solution having ∼300 U/mL of collagenase type II (Worthington), 0.1 mg/mL soybean trypsin inhibitor, and 1 mM CaCl_2_. Every 15 min, the perfusates were collected, centrifuged, resuspended in growth medium, and placed in a 37°C incubator. After coronary perfusate collections were finished for a total of 90 min of digestion, all the resuspended cells were combined into one tube, pelleted by centrifugation, resuspended in plating medium, and plated on 35-mm tissue culture dishes. Diabetic VSMCs were cultured in high-glucose (25 mM) DMEM, whereas nondiabetic VSMCs were cultured in normal-glucose (15 mM) DMEM. All experiments were performed with passage 2 or lower coronary microvascular VSMCs. To obtain adequate numbers of cells for experiments, cells from *n* = 2 mice were pooled into one 35-mm dish for each biological replicate.

### 2.6 Western blot

Human coronary VSMCs were washed twice with ice-cold PBS and then lysed with 90 µL of ice-cold lysate buffer (modified Hunter buffer freshly supplemented with 0.5 mM PMSF, 10 μg/mL aprotinin, 1 mM Na_3_VO_4_). Following sonication at 4°C for 10 s, lysates were spun at 15,000 rpm at 4°C for 15 min. Protein levels were quantified following the specified protocol in the BCA kit (Pierce). Ten micrograms of VSMC protein lysates (*n* = 4-5 per group) were separated on 8%–12% SDS-PAGE gels that were transferred to PVDF membranes for 1 h on ice. The membranes were then blocked in 5% milk in PBS with 0.5% Tween 20 (PBST) at room temperature for 1 h. Antibodies were diluted in 2.5% milk with PBST and then membranes incubated overnight at 4°C. Membranes were washed three times in PBST and then incubated in the appropriate horseradish peroxidase (HRP) secondary antibody for 1 h at room temperature. Membranes were washed again three times in PBST and then imaged and analyzed with Bio-Rad Image Lab.

### 2.7 siRNA and latrunculin B VSMC treatments

Human: Human primary coronary VSMCs (passage 5–7) were plated at 0.4 × 10^4^ cells per well onto an 8-well chamber slide. 24 h later at approximately 60%–80% confluency, cells were then treated for 48 h prior to experiments with either cofilin siRNA (10 µM) or scramble transfection control from Cell Signaling Technology in antibiotic free, low serum DMEM, utilizing Lipofectamine RNAiMAX Transfection protocol following manufacturer’s instructions (Invitrogen/ThermoFisher). Transfection efficiency was confirmed 48 h post transfection by immunofluorescent staining. For Latrunculin B (LatB), cells were incubated in low serum media for 24 h. LatB (1 µM) was added 2 h prior to AFM or fixation.

Mouse: Experiments were performed on passage 1 mouse primary coronary VSMCs. Cells were plated at 0.4 × 10^4^ cells per well onto an 8-well chamber slide. Following 24 h in culture, cells (60%–80% confluency) were treated with antibiotic free, low serum medium containing DharmaFECT 1 transfection reagent and either 100 nmol of nontargeting siRNA or siRNA specifically targeting cofilin (Dharmacon SMARTpool) for 48 h prior to experiments. Transfection efficiency was confirmed 48 h post transfection by immunofluorescent staining. For LatB, cells were incubated in low serum media for 24 h. LatB (1 µM) was added 2 h prior to AFM or fixation.

### 2.8 Atomic force microscopy

For mouse VSMC (n = 5 for all groups) and human VSMC (n = 4-5 for all groups) AFM experiments, cells were treated as described above. Using an Asylum MFP-3D-Infinity-BIO atomic force microscope (AFM) with probes (Bruker MLCT) coated with 0.5 mg/mL fibronectin (FN, Gibco), a nano-indentation protocol (Costa, 2003; Hong et al., 2015) was used to measure elastic modulus, an indicator of cellular stiffness, and adhesion to FN. The following parameters were set for each experiment: Force Distance was set to 1.6 µm, sample rate was set to 625 Hz, and set point was set to 0.3 V. For each experiment, we collected 25–35 curves per cell and 8–12 cells per dish. The AFM data were collected and analyzed using Igor Pro software (WaveMetrics). Elastic modulus was calculated using the Oliver-Pharr method, a modified Hertz model (Akhtar et al., 2009).

### 2.9 Immunofluorescence

Cells were gently rinsed cells with cold phosphate buffer saline (PBS) twice and then fixed on ice with cold 2% paraformaldehyde (PFA) for 30min. After fixation, the cells were rinsed with PBS twice and then treated with PBS-Tween-Triton for a 20-min permeabilization. Next the cells were incubated in fish skin gelatin blocking solution for 30 min at room temperature. Next, for assessing F/G actin, the cells were then incubated for 30 min with DNAse (488) and phalloidin (567), and DAPI to stain for G-actin, F-actin, and the nuclei, respectively. To assess cofilin expression, cells were incubated primary cofilin antibody overnight at 4°. The following morning, cells were washed with PBS three times, then incubated with secondary antibody for 1 h at room temperature. Next, all cells were washed with PBS three times and then fresh PBS was added to cover cells. Cells were then imaged using an Olympus IX51 and intensity per cell was measured using the NIH ImageJ software.

### 2.10 Heart isolation and langendorff perfusion

Isolated mouse whole hearts were rapidly excised and perfused at a constant pressure (80 mmHg) with Kreb’s solution (in mM: NaCl 118, KCl 4.7, MgSO_4_ 1.64, KH_2_PO_4_ 1.18, CaCl_2_ 2.52, NaHCO_3_ 25, Glucose 5.55, Na-pyruvate 2) on a Langendorff apparatus (Model IH-SR, Harvard Apparatus) as previously described by us (Trask et al., 2007; Trask et al., 2008). Flow was continuously monitored using a flow meter (Model TS420, Transonic Flow Systems) connected to a PowerLab 16/30 (AD Instruments), and data were acquired and analyzed using LabChart 8 (AD Instruments). Following a 15-min equilibration, ML-7 (myosin light chain kinase inhibitor to remove basal active tone) was infused directly into the coronary circulation via a custom dual PE-10 catheter using a custom modification to the system’s infusion port that allows for the direct infusion into the coronary circulation. ML-7 was infused toward a target concentration of 50 µM to inhibit MLCK in cells. After 15 min of ML-7, LatB was added to the infusion toward a concentration target of 1 μM, which was shown to effectively inhibit acting polymerization and reduce cellular stiffness (Embry et al., 2016). Infusion rates were controlled using syringe pump (Model 11 Plus, Harvard Apparatus) and were adjusted every 5 min during infusion to account for changes in CBF. The infusion rate was adjusted based on the coronary flow, e.g., at 50 μL/min per coronary flow mL/min (e.g., 50 μL/min when coronary flow was 1 mL/min) for both ML-7 pre-treatment and LatB/ML-7 infusion. Data are reported at 3 timepoints; baseline (after 15-min equilibration/before ML-7 infusion), ML-7 (at the end of 15-min ML-7 pre-treatment), and LatB (at the end of ∼15-min ML-7/LatB infusion).

### 2.11 Statistical analysis

All data are expressed as means ± SEM, with a probability of *p* < 0.05 used to denote statistical significance with GraphPad Prism 8 (GraphPad Software, La Jolla, CA). A power calculation (90% power, α < 0.05), based on mean differences in coronary VSMC stiffness, indicated that *n* = 3 were needed per group. Either ANOVA or Student’s t-test were performed. Data values that were beyond ±2 standard deviations of the mean were excluded from analysis.

## 3 Results

### 3.1 CRM VSCM actin cytoskeleton favors less stiff cell in diabetes

**FIGURE 1:**
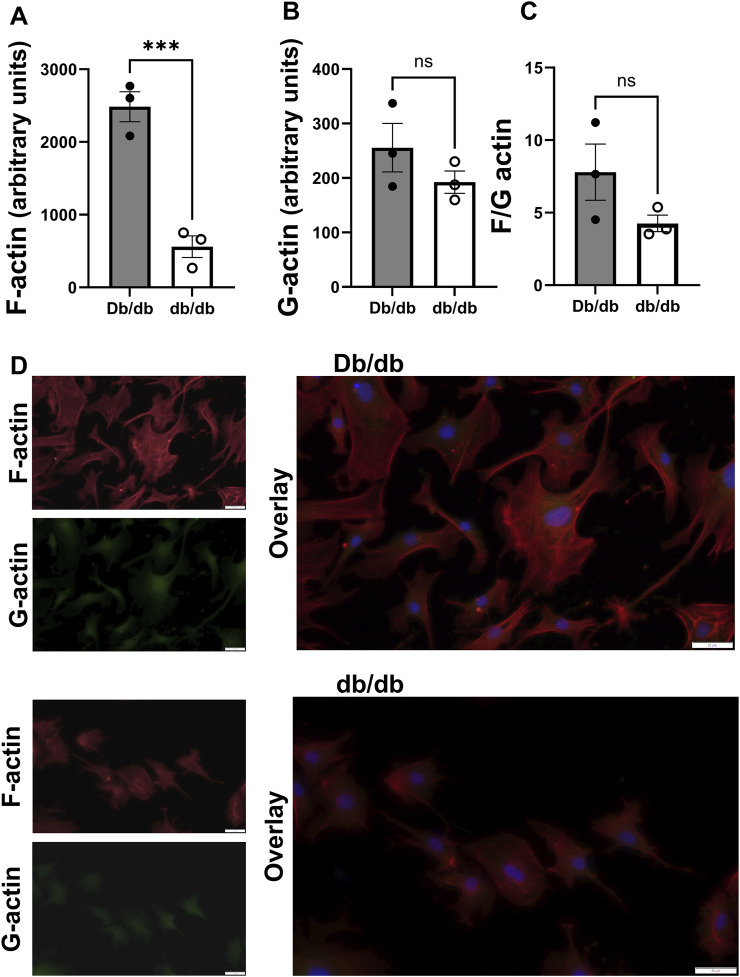
F actin is reduced in diabetic CRMs: **(A)** F actin content in control and diabetic mouse coronary VSMCs obtained via staining with phalloidin. **(B)** G actin levels in control and T2DM mouse coronary VSMCs attained through staining with DNase. **(C)** F/G actin ratio of mouse normal and diabetic coronary VSMCs. **(D)** Representative images from Db/db (top) and db/db (bottom). Data are mean ± SEM. **p ≤ 0.005. n = 3 per group.

Since actin can dynamically polymerize and depolymerize to contribute to the structure and biomechanics of the cell (Serebryannyy et al., 2016), and diabetes can lead to dysregulation of actin polymerization and depolymerization dynamics in other cell types (Romanelli et al., 2020), we aimed to determine whether endogenous F-actin and G-actin content were altered in diabetic db/db mice CRM VSMCs to favor a less stiff cytoskeleton. In mouse primary CRM VSMCs, F-actin content was significantly decreased in db/db CRM VSMCs (normal 2485 ± 206 vs. T2DM 560 ± 149, *p* = 0.0016, n = 3) (Figure 1). There was an insignificant trend for a decreased F/G actin ratio in the diabetic cells (Figure 1). There was no significant difference in G-actin levels between control and T2DM coronary VSMCs (Figure 1).

### 3.2 Cofilin expression is increased in T2DM mouse CRMs and human coronary VSMCs

**FIGURE 2:**
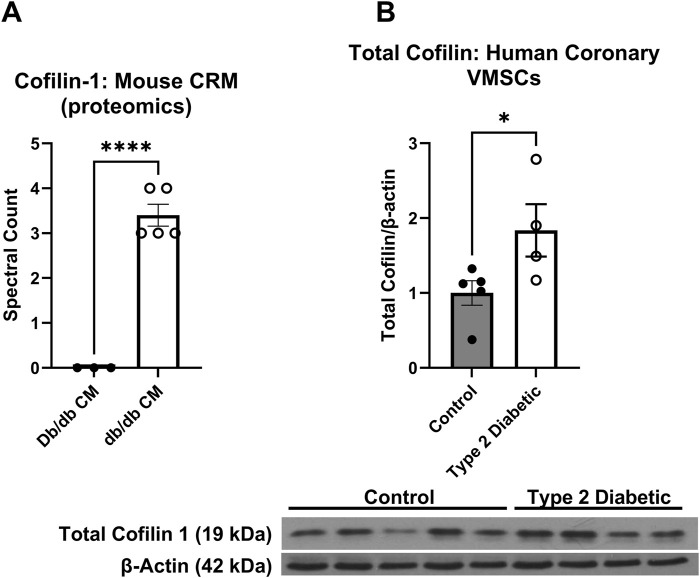
Cofilin is increased in T2DM coronary microvessels: **(A)** Proteomics analysis of cofilin spectral hits in the normal CRMs db/db CRMs. **(B)** Cofilin protein expression obtained via western blot analysis in the coronary VSMCs of normal and diabetic human patients. Data are mean ± SEM. p-values are presented on the graph and *p < 0.05 and ****p ≤ 0.0001. n = 3-5 per group.

Given the well-established role cofilin plays in actin dynamics, we next investigated whether cofilin may account for observed differences in actin cytoskeleton (decreased F-actin) in both diabetic animals and human cells since (Figure 1). Utilizing proteomics analysis, we obtained zero cofilin spectral hits in normal mouse CRMs and 3-4 spectral hits in each db/db CRM pooled sample set (0.00 ± 0.0 vs. 3.40 ± 0.25, p < 0.0001) (Figure 2). In human coronary VSMCs, cofilin protein was increased in the coronary VSMCs of diabetic patients compared to normal (normal 1.00 ± 0.16 vs. T2DM 1.837 ± 0.35, p < 0.05) (Figure 2).

### 3.3 Cofilin knockdown changes F/G actin and stiffness to favor a less stiff cell in human coronary VSMCs

**FIGURE 3:**
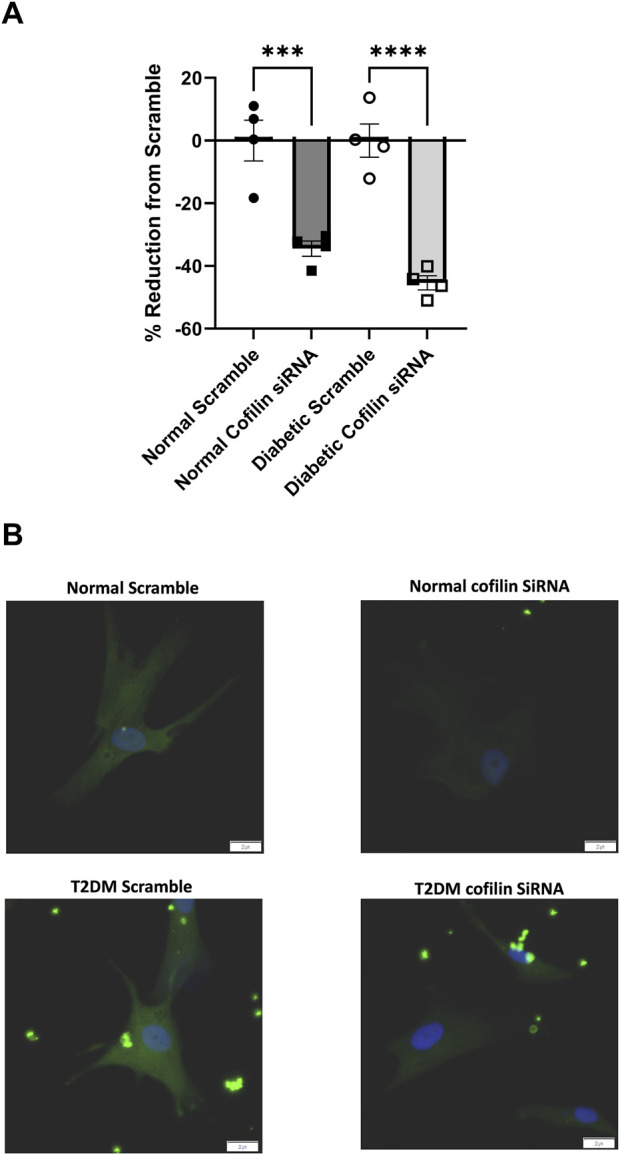
Cofilin siRNA reduces protein expression: **(A)** Percent reduction of cofilin expression after treatment with cofilin siRNA. **(B)** Representative images. Data are mean ± SEM. p-values are presented on the graph; ***p ≤ 0.001 and ****p ≤ 0.0001 vs. respective scramble. n = 4 per group. Scalebar: 20 µm.

**FIGURE 4:**
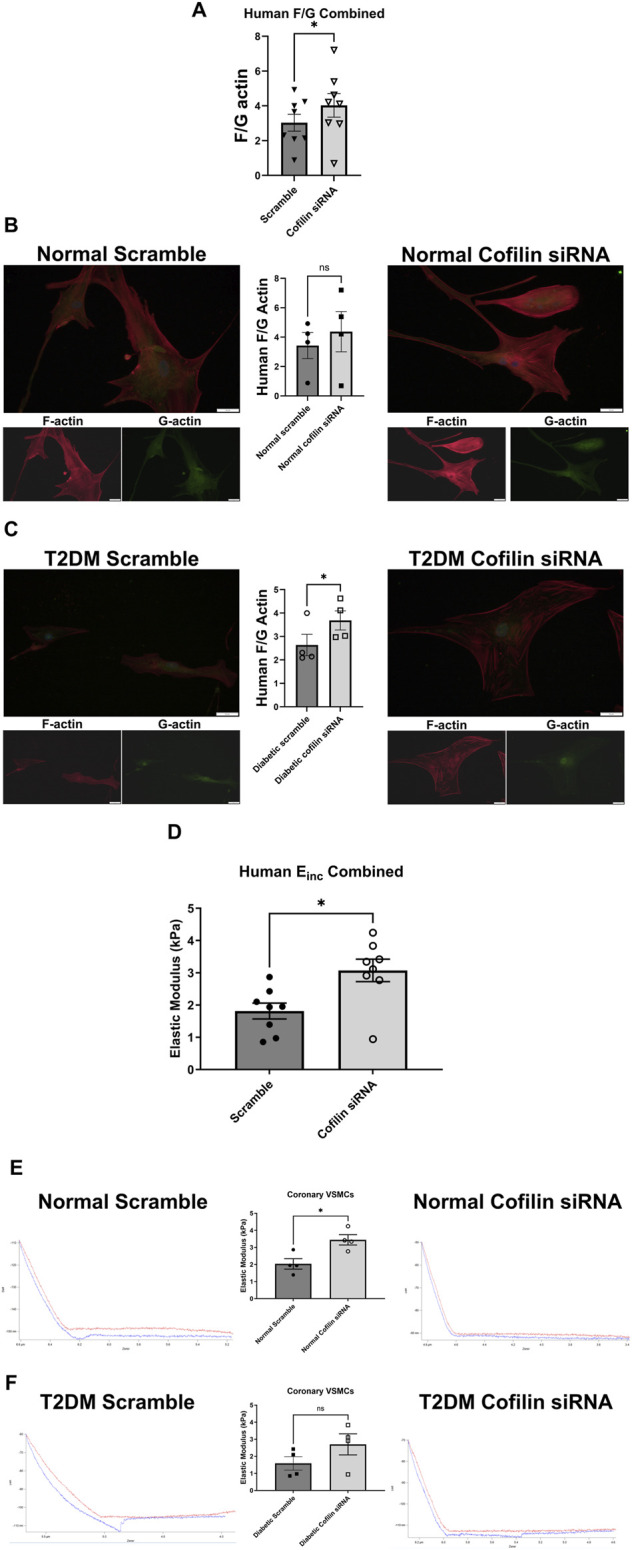
Cofilin knockdown causes altered F/G actin ratio and a significant increase in stiffness in human coronary VSMCs: **(A)** F/G actin ratio of normal and diabetic combined human coronary VSMCs treated with cofilin siRNA obtained via staining F actin (phalloidin) and G actin (DNase). **(B)** F/G actin ratio of cofilin siRNA treated normal coronary VSMCs. **(C)** F/G actin ratio of human diabetic coronary VSMCs treated with cofilin siRNA. **(D)** Elastic modulus (E_inc_) of normal and diabetic combined human coronary VSMCs treated with cofilin siRNA acquired via atomic force microscopy. **(E)** Elastic modulus of cofilin siRNA treated human normal coronary VSMCs. **(F)** Elastic modulus of human diabetic coronary VSMCs treated with cofilin siRNA. Data are mean ± SEM. *p < 0.05. n = 4-8 per group.

Since we observed an increase in cofilin protein in T2DM CRMs and VSMCs, we next interrogated the role of cofilin in VSMC stiffness. We utilized cofilin siRNA to significantly reduce expression (34% reduction in normal, *p* = 0.0190 and 45% reduction in diabetic, *p* = 0.0055) (Figure 3) in our coronary VSMCs. We further tested the contribution of cofilin to the F/G actin ratio of coronary VSMCs by utilizing cofilin siRNA. Cofilin knockdown caused a significant increase in the F/G actin ratio when normal and diabetic data were pooled (scramble 3.029 ± 0.49 vs. siRNA 4.029 ± 0.68, *p* = 0.018, n = 8) (Figure 4). This increased F/G ratio was significant in the diabetic coronary VSMCs (scramble 2.636 ± 0.46 vs. siRNA 3.685 ± 0.41, *p* = 0.049, n = 4), and trended in the normal cells but did not reach statistical significance (scramble 3.423 ± 0.89 vs. cofilin siRNA 4.373 ± 1.37, *p* = 0.22, n = 4) (Figure 4). We next determined how cofilin impacts cellular elastic modulus, an indicator of cell stiffness. Cofilin knockdown by siRNA caused a significant increase in stiffness when normal and diabetic VSMC data were pooled (scramble 1.814 ± 0.25 vs. siRNA 3.074 ± 0.35, *p* = 0.0057, n = 8) (Figure 4) and in normal VMSCs (scramble 2.038 ± 0.31 vs. siRNA 3.445 ± 0.30, *p* < 0.05, n = 4). The trend in the diabetic VSMCs did not achieve statistical significance (scramble 1.589 ± 0.40 vs. siRNA 2.702 ± 0.62, *p* = 0.18, n = 4) (Figure 4).

### 3.4 F/G actin and stiffness are modified via cofilin siRNA in mouse coronary VSMCs

**FIGURE 5:**
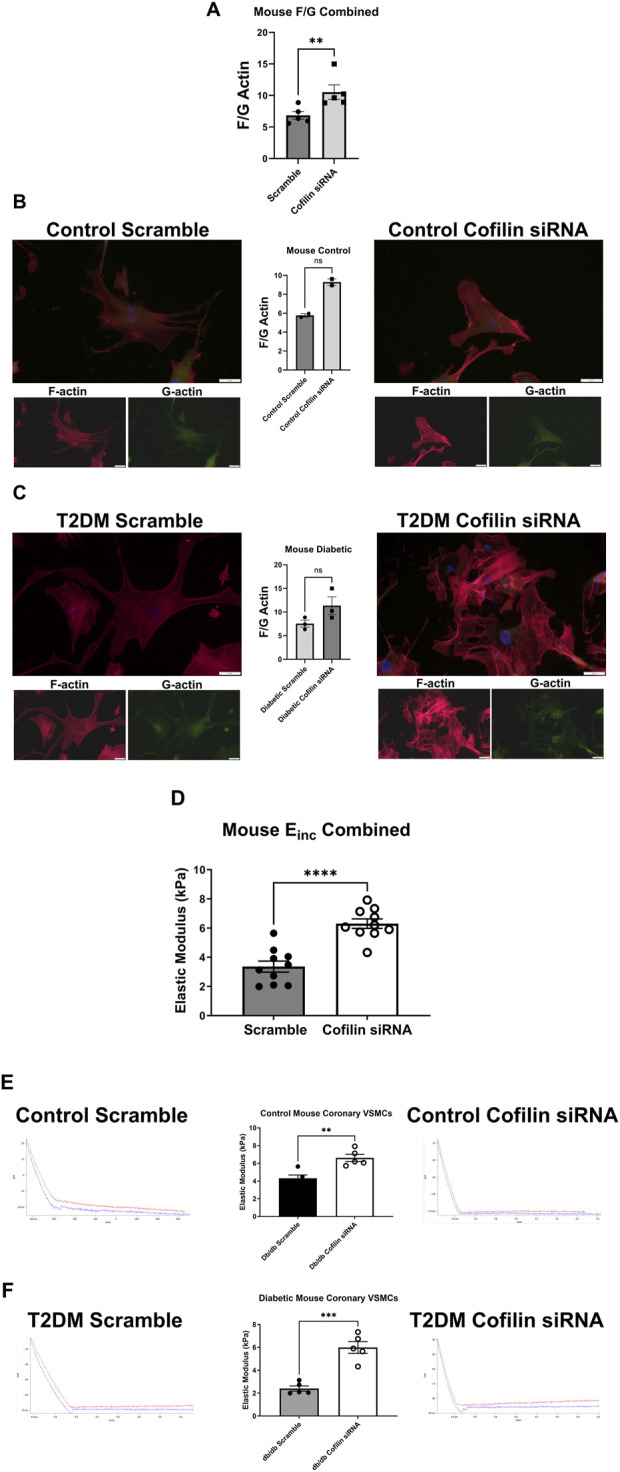
Cofilin siRNA disrupts the F/G actin ratio and increases stiffness in mouse coronary VMSCs: **(A)** F/G actin ratio of mouse normal and diabetic combined coronary VSMCs treated with cofilin siRNA obtained via staining F actin (phalloidin) and G actin (DNase). **(B)** Normal control mouse coronary VSMC F/G actin ratio after cofilin siRNA treatment. **(C)** F/G actin ratio of mouse diabetic coronary VSMCs treated with cofilin siRNA. **(D)** Normal and diabetic combined mouse coronary VSMCs treated with cofilin siRNA elastic modulus (E_inc_) data obtained using atomic force microscopy. **(E)** Elastic modulus of cofilin siRNA treated normal control mouse coronary VSMCs. **(F)** Elastic modulus of mouse diabetic coronary VSMCs treated with cofilin siRNA. Data are mean ± SEM. **p < 0.005, ***p < 0.0005, and ****p < 0.0001 vs. respective scramble control. n = 2–10 per group.

Cofilin siRNA treatment triggered a significant increase in the F/G actin ratio when control and diabetic data were pooled (scramble 6.838 ± 0.60 vs. siRNA 10.53 ± 1.15, *p* = 0.005, n = 5) (Figure 5). This increased F/G ratio trended in the diabetic coronary VSMCs (scramble 7.549 ± 0.74 vs. siRNA 11.35 ± 1.87, *p* = 0.08, n = 3), and trended in the control cells but did not reach statistical significance (scramble 5.772 ± 0.19 vs. cofilin siRNA 9.284 ± 0.34, *p* = 0.09, n = 2) (Figure 5). Cofilin knockdown caused a significant increase in stiffness when normal and diabetic VSMC data were pooled (scramble 1.994 ± 0.38 vs. siRNA 6.303 ± 0.32, *p* < 0.0001, n = 10) (Figure 5). Cofilin siRNA treatment significantly increased control VMSC stiffness (scramble 4.316 ± 0.37 vs. siRNA 6.613 ± 0.40, *p* = 0.003, n = 5) and significantly increased stiffness of the diabetic VSMCs (scramble 2.402 ± 0.23 vs. siRNA 5.993 ± 0.51, *p* = 0.0002, n = 5) (Figure 5).

### 3.5 Latrunculin B significantly reduced VSMC stiffness

**FIGURE 6:**
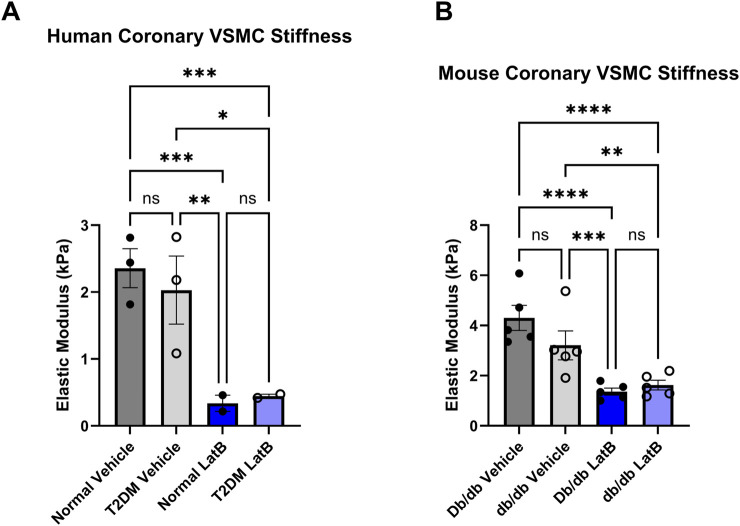
Latrunculin B treatment of coronary VSMCs significantly reduces cellular stiffness: **(A)** Elastic modulus acquired via atomic force microscopy of LatB treated normal and diabetic human coronary VSMCs. **(B)** LatB treated mouse normal and diabetic coronary VSMC elastic modulus. Data are mean ± SEM. *p < 0.05, **p < 0.005, ***p < 0.0005, and ****p < 0.0001. n = 2-5 per group.

Next, we investigated how altering actin with a direct actin-disturbing agent alters elastic modulus. We utilized LatB is a well-known actin depolymerizing agent to disrupt the actin cytoskeleton in coronary VSMCs. In both mouse and human coronary VSMCs, LatB significantly reduced elastic modulus in the normal/control (mouse control vehicle 4.303 ± 1.12 vs. mouse LatB 1.365 ± 0.31, *p* < 0.0001, n = 5 and human normal vehicle 2.357 ± 0.50 vs. normal LatB: 0.3369 ± 0.17, *p* = 0.0003, n = 2–3) (Figure 6) groups as well as in the diabetic groups (mouse T2DM vehicle 3.208 ± 1.13 vs. mouse T2DM LatB: 1.623 ± 0.44, *p* = 0.0048, n = 5 and human T2DM vehicle: 2.030 ± 0.88 vs. human T2DM LatB: 0.4465 ± 0.04, *p* = 0.0200, n = 2–3) (Figure 6).

### 3.6 Latrunculin B normalizes CBF in isolated hearts

**FIGURE 7:**
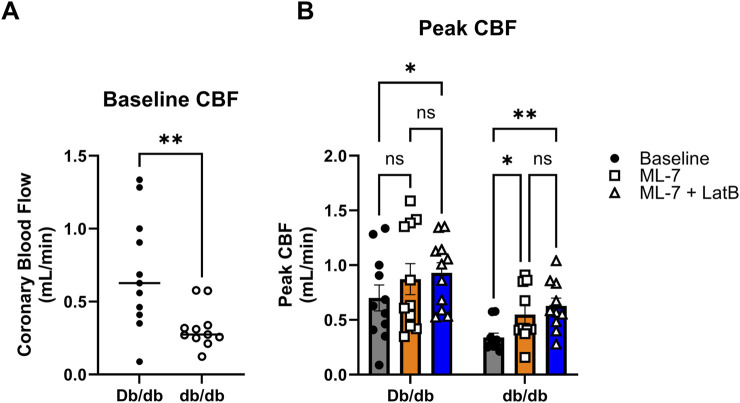
Latrunculin B increases CBF: **(A)** Baseline CBF obtained from normal and T2DM hearts *ex vivo* on the Langendorff perfusion system. **(B)** Peak responses to ML-7 and ML-7 + LatB of *ex vivo* heart on Langendorff perfusion system. Data are mean ± SEM. *p < 0.05, **p < 0.005. n = 11 per group.

To assess whether modulating coronary cell stiffness can affect CBF, we directly infused LatB into the coronary circulation of Langendorff isolated hearts. As we have demonstrated *in vivo* (Katz et al., 2011), baseline CBF in was lower in hearts isolated from T2DM db/db mice (Figure 7A; Db/db: 0.70 ± 0.12 mL/min vs. db/db: 0.32 ± 0.04 mL/min, *p* < 0.01). After baseline measurements, the myosin light chain kinase inhibitor, ML-7, was infused first to remove active coronary tone. ML-7 resulted in a significant increase in CBF in db/db mice (Figure 7B; *p* < 0.05). LatB further augmented CBF in both normal and T2DM hearts (Figure 7B; Db/db: *p* < 0.05 and db/db: *p* < 0.01 vs. respective baselines).

## 4 Discussion

The actin cytoskeleton plays a crucial role in shaping and maintaining the mechanical properties of cells. Its dynamic cycle of polymerization and depolymerization allows cells to adapt to changing conditions in an attempt to maintain their biomechanical functionality. Our laboratory has previously demonstrated that T2DM or MetS coronary resistance microvessels are less stiff compared to normal CRMs, and that coronary VSMCs from T2DM humans and mice have a reduced elastic modulus, the primary indicator of stiffness (Katz et al., 2011; McCallinhart et al., 2020; McCallinhart, Scandling, and Trask, 2021; Trask et al., 2012b). In our current study, we examined how T2DM impacts the actin cytoskeleton, as well as the contribution of the altered cytoskeleton to stiffness and coronary blood flow. We observed significant reduction in F-actin in coronary VSMCs from T2DM mice. Cofilin, an actin depolymerizing protein, was significantly increased in T2DM as validated by both proteomic data from mice and Western Blot data from human coronary VSMCs. The F/G actin ratio was increased when we knocked down cofilin via siRNA treatment, which also led to a concomitant significant increase in VSMC stiffness in both mouse and human coronary VSMCs. We also directly modified the coronary cell actin cytoskeleton utilizing LatB treatment that significantly decreased both mouse and human coronary VSMC stiffness and increased CBF when *ex vivo* hearts were treated with ML-7 (to remove tone) and LatB. These data clearly demonstrate a novel role for cofilin in governing CRM VSMC actin cytoskeleton regulation and further suggest that it may play a role in the regulation of CBF.

T2DM triggers a cascade of biochemical changes that adversely affect the VSMCs cytoskeleton. Actin is most recognized for its role as a key component of the cytoskeleton, where it significantly influences cell shape, movement, and signaling. Diabetes can lead to dysregulation of actin polymerization and depolymerization dynamics (Romanelli et al., 2020). F-actin, the filamentous form, is a major component of the cytoskeleton and is essential for the formation and maintenance of focal adhesions. It is well-documented that the actin cytoskeleton bestows mechanical stability by regulating cell stiffness (Fels et al., 2014; Grimm et al., 2014; Sehgel et al., 2013). We have previously reported that T2DM decreases coronary VMSC stiffness (McCallinhart et al., 2020); as such, we aimed to investigate how the actin cytoskeleton contributes to altered stiffness in coronary VMSCs in the presence and absence of T2DM. Studies have reported that diabetes leads to cytoskeleton disorganization in cardiac and skeletal muscle via measured F-actin fluorescence in muscles of diabetic animals (Romanelli et al., 2020). Similarly, multiple studies have reported decreased F-actin content in diabetic myocardial tissue leading to cytoskeleton disorganization (Kawaguchi et al., 1999; Nemoto et al., 2006; Zhang et al., 2008). Benech et al. (2014) reported that diabetic cardiomyocytes displayed a more diffuse and irregular actin arrangement compared to control isolated cardiomyocytes that exhibited a regular and well-defined actin organization. In the same study, the authors utilized AFM to reveal that both aging and diabetic cardiomyocytes are stiffer compared to controls. Similarly to these studies, we found that control coronary VSMCs have significantly higher F-actin content compared to T2DM coronary VSMCs (Figure 1), which may account for higher stiffness in normal CRMs compared to T2DM CRMs (Katz et al., 2011; McCallinhart et al., 2020; Trask et al., 2012a).

To interrogate a possible mechanism that may be responsible for reduced F-actin content in the diabetic coronary VSMCs, we focused on cofilin initially based on a proteomic screen (Figure 2). Cofilin is an endogenous actin regulatory protein that depolymerizes F-actin and severs the actin filaments. While very little is known about cofilin in the coronary VSMCs, actin dynamics are crucial in many organs and tissues in T2DM, including a role for cytoskeletal remodeling in maintaining neuronal morphology and long-term memory (Lacolley et al., 2017). Interestingly, diabetic mice exhibited cognitive impairment at 17 weeks of age and cofilin and G-actin were highly expressed in the CA1 region of hippocampus, while phosphorylated (P)-cofilin and F-actin expression decreased (Li et al., 2023). Other recent studies have highlighted the role of cofilin in actin dynamics in diabetic conditions. Hyperglycemic conditions stimulated actin polymerization measured via F/G actin ratio and induced mRNA and protein expression of contractile smooth muscle markers in cultured mouse aortic VSMCs (Hien et al., 2016). In that same study, cofilin phosphorylation was significantly increased via high glucose treatment (Hien et al., 2016). In this study, we showed that total cofilin levels were significantly increased in T2DM human coronary VSMCs, as well as in the CRMs of db/db T2DM mice (Figure 2). These studies implicate a role for glucose levels impacting functionality and cofilin and the actin cytoskeleton.

Given the well-established relationship between cofilin and its regulation of actin remodeling, we next examined how knocking down cofilin would impact the F-actin to G-actin ratio in coronary vascular smooth muscle cells. In both human and mouse coronary VSMCs, cofilin siRNA significantly increased the F/G actin ratio when normal and diabetic data are combined (Figures 4, 5). This trend persisted when normal and diabetic are separated but only reached significance in the human coronary diabetic VSMCs (Figure 4). Interestingly, recent studies have shown that portions of F-actin bound to cofilin have a decreased stiffness compared to their unbound F-actin counterparts (McCullough et al., 2008; Schramm et al., 2017). These data are quite intriguing given our previous data of decreased elastic modulus in primary diabetic coronary VSMCs and increased cofilin protein expression in human coronary VSMCs and mouse CRM tissue (Figure 2) (Katz et al., 2011; McCallinhart et al., 2020; Trask et al., 2012b). To investigate the role of cofilin in the stiffness of coronary VSMCs, we utilized AFM to measure elastic modulus, an indicator of cellular stiffness. Both cofilin siRNA treated mouse and human coronary VSMCs showed a significant increase in elastic modulus, likely due to the increased F/G actin ratio (Figures 4, 5). In the mouse coronary VSMCs, both normal and diabetic elastic modulus was significantly increased when the cells were treated with cofilin siRNA (Figure 5). Other studies have implicated a role for cofilin in vascular mechanics in disease. For example, LIM Kinase (LIMK) is an enzyme that inactivates cofilin via phosphorylation. Intriguingly, inhibiting LIMK prevented small arteriolar agonist-induced inward remodeling implicating cofilin and actin polymerization play a role in microvascular remodeling (Foote et al., 2016). Morales-Quinones et al. reported that localized LIM kinase inhibition prevents arteriolar inward remodeling in hypertensive mice suggesting that hypertension may be correlated with phosphorylation of VSMC cofilin and actin stress fiber formation leading to heightened arterial stiffness (Morales-Quinones et al., 2020). In this study, we observed increased cofilin in T2DM CRM VSMCs (Figure 2), and cofilin knockdown significantly increased coronary VSMC stiffness (Figures 4, 5). We postulate this is due to cofilin’s role in transforming the actin cytoskeleton (Figures 4, 5). Collectively, these data warrant further investigation into the role for LIMK and cofilin in coronary vascular mechanics.

Altering the cytoskeleton via pharmacological agents such as cytochalasin B and LatB causes a striking reduction in cell stiffness in mouse 3T3 fibroblasts and chicken embryo fibroblasts, respectively (Petersen et al., 1982; Wakatsuki et al., 2001). In isolated adult cardiomyocytes, Wu and colleagues showed that cytochalasin D and 2,3-Butanedione monoxime (BDM) treatments decreased cell stiffness by 70%–85% supporting that cardiomyocyte stiffness is dependent on the actin-myosin cytoskeleton (Wu et al., 2010). In our study, LatB treatment drastically reduced normal and diabetic coronary VSMC stiffness in both human and mouse cells (Figure 6). Utilizing AFM and fluorescent actin imaging, Gavara et al. (Gavara and Chadwick, 2016) determined that increasing amounts of actin in stress fibers caused increase in cell stiffness. Altering the actin cytoskeleton in the cells of vessels also impacts whole vessel tension. Treatment of the pulmonary artery and aorta *ex vivo* with cytochalasin D to depolymerize actin profoundly inhibited vessel tension development (Adler et al., 1983; Wright and Hurn, 1994). Conversely, actin stabilizers/polymerizers, including jasplakinolide, enhance myogenic tone via smooth muscle contraction and decreased globular- (G) to filamentous- (F) actin ratio (Cipolla et al., 2002). These actin-altering drugs are great tools to explore the balance between the filamentous and globular forms of actin and their role in cytoskeletal balance and function.

Given their roles in regulating the actin cytoskeleton, several reports have explored the potential mechanistic association of Latrunculin A, and B, and cofilin. Cofilin 1 contributes to fast, rapid actin dynamics by depolymerizing actin filaments. Interestingly after a 5-min Latrunculin A treatment, in B16F1 cells pre-treated with cofilin 1 siRNA, only ∼10% of the cells had lost their stress fibers and after 30-min Latrunculin A treatment a substantial amount of these cells (roughly 80%) were morphologically approximately normal (Hotulainen et al., 2005). This implicates a competing mechanism of cofilin and Latrunculin-A on the alteration of the actin cytoskeleton. Jovceva et al. (Jovceva et al., 2007) utilized LatB to sequester G-actin in S2R + cells, leading to a continuous increase in the phosphorylation of cofilin in a short 30–60-min period and caused the rapid loss of cortical F-actin. Conversely, treatment with jasplakinolide, a drug that promotes actin filament formation, generated the rapid loss of P-cofilin and was also able to block the accumulation of P-cofilin in insulin-treated cells (Jovceva et al., 2007). Interestingly, in that same study, they reported that in S2R + cells, slingshot (ssh) RNAi suppressed both jasplakinolide- and Latrunculin-induced changes in P-cofilin rather than LIMK RNAi, a well-known regulator of cofilin. Short term treatment of myoblasts with insulin led to rapid cofilin dephosphorylation, however, this dephosphorylation was prevented with LatB treatment (Chiu et al., 2010). Similarly, treatment of MCF-7 cells with Latrunculin A suppressed SSH1L-induced cofilin dephosphorylation (Nagata-Ohashi et al., 2004). In STHdh cells, a specific cell generated to study Huntington disease, LatB treatment initiated co-localization of cofilin-labeled rods and phalloidin (F-actin) (Serebryannyy et al., 2016). In peritoneal mast cells, LatB treatment increased g-actin levels and caused translocation of both actin and cofilin into the nuclei (Pendleton et al., 2003). Further, utilizing anti-cofilin antibody on permeabilized cells reduced nuclear actin accumulation, demonstrating the dependence on cofilin for actin nuclear translocation (Pendleton et al., 2003). Collectively, these studies implicate a competing mechanism for cofilin and LatB.

Coronary blood flow (CBF) delivers essential nutrients to cardiac tissue, supporting its metabolic need. The coronary vessels are responsible for carrying blood to and from the cells of the heart, so any disruption in CBF can result in significant health issues, including heart failure. Previously, our group demonstrated that inward hypertrophic remodeling of CRMs was an early contributor to CAD and was associated with reduced coronary flow in both the db/db mouse model of T2DM and a Ossabaw swine model of MetS (Katz et al., 2011; Trask et al., 2012a). We believe this reduced T2DM stiffness is a compensatory mechanism to maintain blood flow to the myocardium; however, the association of coronary microvascular stiffness as it relates to coronary blood flow has never been explored. There is evidence that arterial stiffening adversely affects coronary flow and flow reserve, but those data are taken from large conduit arteries outside of the coronary circulation (Fukuda et al., 2006; Leung et al., 2006; Tritakis et al., 2016). We have previously reported that in keeping with Poiseuille’s Law (flow is proportional to radius to the 4^th^ power), we observed that decreased CRM diameter was associated with reduced coronary blood flow (Katz et al., 2011; McCallinhart et al., 2021; Trask et al., 2012b). We have also previously reported reduced stiffness, however, it is plausible that in the absence of a reduction in CRM VSMC stiffness that coronary blood flow would be further reduced (McCallinhart et al., 2021). Therefore, the reduction in CRM VSMC stiffness may be a compensatory mechanism to limit reduction in coronary blood flow in T2DM. To assess this possibility, we aimed to uncover the regulation of the cytoskeleton and cell stiffness in CRM VSMCs, and ultimately, how it impacts CBF. The treatment of *ex vivo* vessels with pharmacological actin-targeting agents impacts vessel tension and stiffness. Cytochalasin D, an inhibitor of actin polymerization, was reported to inhibit pulmonary artery and aorta vessel tension development (Adler et al., 1983; Wright and Hurn, 1994). On the contrary, the actin polymerizer jasplakinolide enhanced myogenic tone via smooth muscle contraction and decreased G-actin content (Cipolla et al., 2002). In this study, we treated coronary VSMCs with LatB to depolymerize the actin cytoskeleton and drastically decreased elastic modulus (Figure 6). To test whether these observations held true in an intact heart and to further determine their impact on CBF, we employed the classical Langendorff isolated heart technique. This technique allows for the direct assessment of isolated CBF without significant confounding variables that accompany *in vivo* assessments. We directly infused *ex vivo* hearts with ML-7 and LatB on a Langendorff perfusion system to determine how altering cellular stiffness impacts CBF. ML-7 is a well-documented myosin light chain kinase inhibitor. Myogenic tone and vascular stiffness both contribute to vascular biomechanics and function but have different origins. Myogenic tone is partial constriction of a blood vessel that is triggered by changes in blood pressure via calcium-induced contraction of the actin-myosin myofilament apparatus in VSMCs. Previous studies have documented an augmented myogenic response in the coronary circulation of diabetic animals (Belmadani et al., 2008; Moien-Afshari et al., 2008; Trask et al., 2012a). These dilations and constrictions of the actin-myosin apparatus during myogenic tone alter CBF in a short-term dynamic manner whereas vascular stiffness alters CBF in a long-term manner. To eliminate these short-term changes and the role of myogenic tone, and to solely focus on how changes in the actin cytoskeleton (cellular stiffness) impact CBF, we pretreated the *ex vivo* hearts with ML-7. Interestingly, upon treatment with ML-7 alone, the peak CBF response of diabetic hearts over baseline increased significantly (Figure 7). It is tempting to speculate that this finding could be due to the aforementioned enhanced basal myogenic responsiveness in T2DM CRMs relative to control. Concomitant treatment of ML-7 and LatB resulted in significant increases in peak CBF of both control and diabetic hearts over baseline (Figure 7). These data demonstrate that the reduction of the F/G actin ratio and cell stiffness via treatment with LatB results in an inversely related increase in CBF (Figure 7). This further supports our notion that in the T2DM hearts, the reduced CRM stiffness is a compensating mechanism to attempt to maintain functional CBF in the heart. This is the first study to investigate the relationship between actin cytoskeleton altering drugs and CBF *ex vivo*.

## 5 Conclusion

In this study, we confirm in both mouse and human coronary vascular smooth muscle a reduction in F-actin in diabetic cells, corresponding to lower stiffnesses. We also demonstrate that the actin regulatory protein, cofilin, is upregulated in diabetic coronary microvascular tissues and smooth muscle cells and knocking it down using siRNA increases the F/G actin ratio and incremental modulus. We also show that pharmacological depolymerization of the actin cytoskeleton using latrunculin B reduces primary coronary VSMC stiffness *in vitro* and that LatB infused directly into the coronary circulation of isolated hearts increased coronary blood flow. These data are the first demonstration in any vascular bed that direct modulation of vascular cell stiffness in the absence of tone can inversely modulate blood flow, and it provides a novel mechanism of blood flow regulation. In summary, this study demonstrates a novel role for cofilin in the cytoskeletal and mechanical regulation of coronary VSMCs, and we further demonstrate a novel inverse relationship between coronary cell stiffness and CBF that has the potential to be exploited for the development of novel therapeutic targets.

## Data Availability

The original contributions presented in the study are included in the article/supplementary material, further inquiries can be directed to the corresponding author.

## Abbreviations

AFM, atomic force microscopy; CAD, coronary artery disease; CBF, coronary blood flow; CMD, coronary microvascular disease; CRM, coronary resistance microvessel; ECM, extracellular matrix; LatB, latrunculin B; MetS, metabolic syndrome; MI, myocardial infarction; T2DM, type 2 diabetes mellitusv; VSMC, vascular smooth muscle cell.
